# Nurses Who Are More Willing to Participate in the Fight against COVID-19: Evidence from China

**DOI:** 10.3390/ijerph18147357

**Published:** 2021-07-09

**Authors:** Lai-Kun Tong, Ming-Xia Zhu, Si-Chen Wang, Pak-Leng Cheong, Iat-Kio Van

**Affiliations:** 1 Research Management and Development Department, Kiang Wu Nursing College of Macau, Macau 999078, China; chingco@kwnc.edu.mo; 2 Education Department, Kiang Wu Nursing College of Macau, Macau 999078, China; zmx@kwnc.edu.mo (M.-X.Z.); sichen@kwnc.edu.mo (S.-C.W.); joecheong@kwnc.edu.mo (P.-L.C.)

**Keywords:** COVID-19, nurse, willingness

## Abstract

When facing an infectious disease disaster, nurses’ willingness to work is critical. Nurses’ lack of willingness to work during a pandemic may worsen the shortage of health care personnel. The purpose of this study is to assess the willingness of nurses to participate in the fight against COVID-19 in China and to identify factors associated therewith. This cross-sectional study examines nurses working in 11 Chinese cities including Macau, Hong Kong, Shenzhen, Dongguan, Huizhou, Guangzhou, Zhaoqing, Foshan, Jiangmen, Zhongshan, and Zhuhai. Questionnaires were collected from 19 May to 7 August 2020. A total of 8065 questionnaires were received, of which 8030 valid questionnaires were included for analysis. A total of 53.4% of participants reported that they had signed up to support the COVID-19 pandemic response. Multivariate logistic regression analysis revealed that being single (OR = 0.72, 95% CI: 0.60–0.87), having no children (OR = 0.81, 95% CI: 0.68–0.97), possessing higher professional qualifications (OR = 1.25, 95% CI: 1.14–1.37), having a more prestigious professional title (OR = 1.68, 95%CI: 1.50–1.90), being an administrative supervisor (OR = 0.53, 95% CI: 0.45–0.63), having a higher caring dimensions inventory score (OR = 1.01, 95% CI: 1.01–1.01), working in a hospital (OR = 0.53, 95% CI: 0.39–0.72), and receiving employer-provided care training (OR = 0.77, 95% CI: 0.68–0.87) were predictive of nurses’ willingness to participate in the fight against COVID-19. We suggest that unmarried nurses should be given priority when recruiting to fight an epidemic and, for married nurses with children who are recruited to fight an epidemic, supporting measures should be provided for childcare. We suggest strengthening workplace training of caring for nurses in order to better retain and recruit qualified support for an epidemic outbreak of infectious diseases.

## 1. Introduction

The coronavirus disease 2019 (COVID-19) pandemic, caused by the severe acute respiratory syndrome coronavirus 2 (SARS-CoV-2) [1], has caused more than 1.1 billion infections and nearly 2.7 million deaths worldwide, as of 15 March 2021 [2]. The prevalence of SARS-CoV-2 infection among health care workers (HCW) in different regions ranged from 0.4% to 57.06% and the estimated pooled prevalence was 11% [3]. Nurses may be more affected by the COVID-19 outbreak than other HCW, with higher rates of SARS-CoV-2 infection and psychological distress [3,4].

Nurses are the largest cadre of the HCW in all countries and play an important role in the response to a global pandemic [5]. Before the outbreak of COVID-19, there had been an extant, global shortage of nurses, and the World Health Organization had recommended that health care providers develop plans to cope with this shortage [6]. Nursing managers during the COVID-19 crisis mitigated for this shortfall by recruiting volunteers and temporary nurses [7]. When facing an infectious disease disaster, nurses’ willingness to work is critical, yet the percentage of nurses willing to work during the last influenza pandemic ranged from 23.1% to 90.1%, depending on their social and professional contexts [8].

Nurses’ lack of willingness to work during a pandemic may worsen the shortage of this profession. Effective preparation for the next pandemic requires assessing the willingness of nurses to have participated in the fight against COVID-19 and understanding the factors that influenced such willingness. Several studies conducted during the COVID-19 pandemic have shown that age, marital status, area of work, level of knowledge, positive professional perception, communication from managers, and risk category may be predictors of willingness [9,10,11,12].

All nurses, including general, specialist, hospital, and community nurses are the backbone of the fight against COVID-19 [13]. However, the small sample size of our studies, or the fact that participants were selected from the same institutions or from within specific groups of nurses (e.g., nurse practitioner) limited the generalizability of our results. However, Chinese nurses’ participation in the fight against COVID-19 was not restricted by region. For example, more than 42,000 medical workers from across China travelled to Hubei Province to combat the epidemic [14]. Samples in this study were collected from various cities and institutions, including hospitals and community institutions, and the sample size was increased to improve sample representativeness. Existing studies have only asked participants about their willingness to work during COVID-19; however, these studies did not further explore whether their participants had indeed participated. During the COVID-19 pandemic, governments and medical institutions around the world recruited nurses to fight the disease. Therefore the willingness of nurses to participate in an epidemic is of global significance, and can be better understood by investigating whether reported willingness to work was congruent with behavior during COVID-19.

The purpose of this study is to assess the willingness of nurses to participate in the fight against COVID-19 and to identify its associated factors.

## 2. Materials and Methods

### 2.1. Ethic

This study obtained ethical approval from the Research Management and Development Department of Kiang Wu Nursing College of Macau (reference no: 2019APR01). Participants were required to give informed consent before filling completing our questionnaire. The survey was conducted anonymously. Participants could withdraw from the research or exit the survey at any time. The collected data were stored on encrypted computers and online platforms.

### 2.2. Study Design and Sample

Ours is a cross-sectional study conducted from 11 Chinese cities including Macau, Hong Kong, Shenzhen, Dongguan, Huizhou, Guangzhou, Zhaoqing, Foshan, Jiangmen, Zhongshan, and Zhuhai. There were 258,364 nurses in these cities. To increase the survey representativeness, the sample size was set at 3% of the nursing population, producing a required sample size of 7751.

The inclusion criteria were employment as a nurse, (accredited by the local government) in hospitals, clinics, schools, services for the elderly, etc. in the above cities, and having obtained qualification as a nurse practitioner. The exclusion criteria for this study were employment as trainees or probationary nurses, and nurses who were unwilling to participate.

### 2.3. Measures

The online questionnaire included four parts: (1) socio-demographic, professional information, and workplace information of the participants; (2) the caring dimensions inventory; (3) the nurse’s career identity scale; and (4) willingness to participate in the fight against COVID-19.

Part 1: General information. Part 1 included questions about socio-demographic data (such as gender, age, marital status, number of children, etc.), profession and workplace-related characteristics (such as professional qualifications, professional title, professional experience, being an administrative supervisor, having received care training in college, having worked at least one year in the nursing profession and/or their present organization, type of health facility, adequacy of their regional workforce, and having received employer-provided care training, etc.) of the participants.

Part 2: The caring dimensions inventory. The caring dimensions inventory (CDI) consists of 25 items, each assessed on a 5-point Likert scale (1 = strongly disagree, 5 = strongly agree) developed by Watson and Lea to measure perceptions of caring [15], with scores ranging from 25 to 125. It is cross-culturally applicable [16,17]; the original English inventory was translated into Chinese using a systematic translation process which included a forward-backward translation, consensus meeting, pilot testing, and a psychometric analysis [18]. The Cronbach’s α coefficient and the content validity index (CVI) of the Chinese version inventory were 0.97 and 0.98 respectively [19].

Part 3: The nurse’s career identity scale. The nurse’s career identity scale (NCI) consists of 13-item, each assessed on a 7-point Likert scale, with scores ranging from 1 to 7, where higher scores indicate a higher level of professional identity. The Chinese version of the NCI was modified by Zhao et al. [20]. Good reliability (Cronbach’s α = 0.84) and validity (CVI = 0.92) of NCI in the Chinese version were demonstrated [20].

Part 4: Intention to participate in the fight against COVID-19. Willingness to participate in the fight against COVID-19 was assessed with a single question: “Have you registered to support COVID-19 pandemic response?”

### 2.4. Data Collection Procedure

The research team set up a liaison system in 11 cities, with a volunteer from hospitals, universities or nursing professional societies in each city to promote the survey in their respective cities. Participants could enter the electronic questionnaire platform with the QR code or URL on our recruitment poster to be presented with an informed consent procedure and, thereafter, complete the questionnaire. The electronic questionnaire was designed to be completed only once per device to avoid duplicate data. The questionnaires were collected from 19 May to 7 August 2020.

### 2.5. Data Analysis

Continuous variables were reported as mean ± standard deviation and a *t*-test was used for comparison between groups. Frequencies and proportions were used for categorical variables and comparison between groups was performed using chi-squared tests or Fisher’s exact test. Multivariable binary logistic regression (forward stepwise likelihood ratio method) was employed to identify the predictors for nurses’ willingness to participate in the fight against COVID-19. Statistical analyses were performed using SPSS 22.0 software (SPSS Inc., Chicago, IL, USA). The threshold for statistical significance was set to 0.05.

## 3. Results

### 3.1. Characteristics of Participants

A total of 8065 questionnaires were received, of which 8030 valid questionnaires were included in the analysis. The mean age of the participants was 31.9 years (SD = 8.7). The majority were female (96.6%), married (63.9%), and held a bachelor’s degree (52.8%). More than 97% of participants were working in hospitals. Among the participants analyzed, approximately 70% had junior professional titles, with a mean professional experience of 11.5 years (refer to Table 1 for details).

### 3.2. Nurses’ Willingness to Participate in the Fight against COVID-19

A total of 53.4% of participants reported that they had registered to support the COVID-19 pandemic response. The results of the individual factors associated with nurses’ willingness to participate in the fight against COVID-19 are shown in Table 1. The results show that no statistically significant difference in participatory willingness was found by gender composition within the workforce. Those who did not show willingness to participate in the fight against COVID-19 were more likely to be younger, married, have children, have lower professional qualifications or title, be in a role other than administrative supervisor, have less profession experience, have lower CDI and/or NCI scores, work in community health facilities, and not have received care training from their employer than those who were willing to participate in the fight against COVID-19.

### 3.3. Predictors of Nurses’ Willingness

The Hosmer–Lemeshow test showed that there was no significant difference between the observed and predicted probabilities of the binary logistic regression model (χ2 = 10.667, *p* = 0.221), indicating good model fit. Regarding socio-demographic factors, only marital status and parenthood were predictors of the willingness to participant in the fight against COVID-19. Unmarried nurses (OR = 0.72, 95% CI: 0.60–0.87) and those having no children (OR = 0.81, 95% CI: 0.68–0.97) were more willing to participate than those in marriages or with children. Regarding professionally related factors, nurses with higher professional qualifications (OR = 1.25, 95% CI: 1.14–1.37), a higher professional title (OR = 1.68, 95% CI: 1.50–1.90), and a higher CDI score (OR = 1.01, 95% CI: 1.01–1.01) were associated with higher willingness to participate in the fight against COVID-19. Nurses who were administrative supervisors (OR = 0.53, 95% CI: 0.45–0.63) were also more willing to participate in the pandemic response than those who were not. Regarding the influence of workplace-related factors on willingness, multivariate logistic regression analysis revealed that nurses who worked in hospitals (OR = 0.53, 95% CI: 0.39–0.72) and whose employers provided care training (OR = 0.77, 95% CI: 0.68–0.87) were more likely to participate in the fight against COVID-19 than those who worked in the community and/or whose employers did not provide such training. (Table 2).

## 4. Discussion

Previous surveys have shown that 61–97% of some nursing populations are willing to participate in the fight against COVID-19 [9,10,11,21,22], while the results of this study show that less than 55% of Chinese nurses have signed up to support the COVID-19 pandemic response. More than 3% of nurses sent by the Chinese government to Wuhan (where the first COVID-19 case was reported) to join the battle against COVID-19 expressed their unwillingness to participate in frontline work during the COVID-19 outbreak [22]. Meta-analysis found that the pooled prevalence of anxiety, depression, and sleep disorders among nurses during the COVID-19 pandemic was 37%, 53%, and 43%, respectively [23]. Nearly 25% of nurses in one hospital reported they intended to leave the field of nursing after the COVID-19 pandemic [24]. As the COVID-19 pandemic continues to develop, the shortage of nurses has worsened. Managers must involve nurses in the response to the epidemic to alleviate workforce shortages. An Australian study has suggested that strategies to improve knowledge of the COVID-19 pandemic, preparedness of the intensive care unit, and personal concern are ineffective in promoting nurses’ active participation in fighting the epidemic, and that communication from managers is most effective [9].

This survey found that gender and age were not associated with nurses’ willingness to participate in the fight against COVID-19, which was consistent with the findings in other studies [9,10]. In addition, some studies showed that male and young HCW were more willing to participate in epidemic work [25,26]. This finding was not replicated, and may be explained by the proportion of male nurses in China, which is approximately 2% of the nursing workforce [27], while this proportion is much higher in other countries. Univariate analysis showed that younger nurses had a higher willingness to be participatory, but multivariate analysis showed that age was not a predictor after controlling for other factors. As in previous research, this study found that married nurses were less likely than nurses who were single to participate in the COVID-19 pandemic response, especially those who were married with children [26,28,29]. One possible reason may be that women were the main family caregivers in the observed populations, and they bore more responsibility at home in addition to their professional responsibilities. Surveys conducted by Mattingly et al. [30] and Craig [31] showed that, in some populations, compared with fathers, mothers were expected to spend more time with their children and have more overall responsibility for managing care. It is suggested that unmarried personnel should be given priority when recruiting during an epidemic, and that, to recruit married nurses who are also parents, supporting measures should be provided for childcare.

Previous studies have been inconsistent regarding the association of professional experience with nurses’ willingness to participate in fighting the epidemic [12,32]. The results of multiple logistic regression analysis showed that a predictor was not professional experience, but rather professional title. It was predictive of Chinese nurses’ attitudes towards dying patients, while clinical experience was not [33]. Nurses with more highly-regarded professional titles had a more positive attitude towards dying patients [33]. The incidence of case fatality rates for COVID-19 in China was 3.8% compared with 14.6% between January and April 2020 [34]. This means that nurses participating in the COVID-19 pandemic response are more likely to face caring for dying patients. However, Chinese nurses lack hospice care education and training [35]. The lack of knowledgeability surrounding hospice care was seen to put pressure on nurses caring for COVID-19 patients [36] and reduce their willingness to participate in the fight against COVID-19. As a result, we suggest medical institutions seeking to retain qualified personnel during a pandemic should strengthen nurses’ training in hospice care.

Although professional esteem for nurses increased during the COVID-19 epidemic [37], the results of multiple regression in this study showed that the more significantly influencing factor of nurses’ willingness to participate in the fight against the epidemic was not professional identity but perceptions of caring. Care is an important part of nursing; training in caring can improve nurses’ mentality surrounding care. Additionally, the results of this study showed that the factor affecting nurses’ willingness to participate in the fight against COVID-19 was not whether they had received relevant training at all during their educations, but whether their working institutions provided relevant training afterward. Nurses with no care training were 77.0% less likely to participate in the epidemic response than those with training provided by their workplaces. It is suggested that at-work institutional training is more important than academic training for improving the caring mentality of nurses. One explanation may be that, while schools do seek to develop nursing students’ caring abilities, the academic process may undermine the students’ valuation of care [38]. Thus, the development of caring abilities may depend upon in-service training. Less than three quarters of the nurses surveyed had received workplace training in care. We therefore suggest strengthening workplace training of caring for nurses to decrease professional reluctance during an outbreak of infectious diseases.

This present study had limitations. First, although the sample size was 3% of the total nursing population and the backgrounds of participants were matched to the population as much as possible, the sample was not randomly selected, so the representativeness of the sample was correspondingly limited. A second limitation was the cross-sectional nature of this study; it is not possible to draw causal inferences between our identified factors and nurses’ willingness to participate in a pandemic response. Third, the use of the Student’s *t*-test for between-group comparison in such a large sample was not optimal because it is sensitive to population size.

## 5. Conclusions

More than half of the respondents in this survey were willing to participate in the fight against COVID-19. The nurses who were married, had children, worked in the community, or were not administrative supervisors reported less willingness to participate in the fight against COVID-19, while those with higher education, professional titles, and CDI scores, or workplace training in caring, had a greater willingness to participate. It is suggested that unmarried nurses should be given priority when recruiting to fight an epidemic. When married nurses with children are recruited, supporting measures should be provided for childcare. Finally, we suggest strengthened workplace training of caring for nurses to better retain and recruit qualified support during the outbreak of infectious diseases.

## Figures and Tables

**Table 1:** Characteristics of participants and their willingness to participate in the fight against COVID-19 (*n* = 8030).

Demographics	Categories	Oversll, *n* (%)/Mean (SD)	Not Registered,	Registered,	χ^2^/t	*p*-Value
			*n* (%)	*n* (%)		
Socio-demographic characteristics						
gender					3.244	0.072
	male	274 (3.4)	113 (41.2)	161 (58.8)		
	female	7756 (96.6)	3627 (46.8)	4129 (53.2)		
age *		31.9 (8.7)	31.4 ± 8.7	32.3 ± 8.6	−0.063	0.000
marital status					14.320	0.001
	single	2741 (34.1)	1213 (44.3)	1528 (55.7)		
	married	5127 (63.9)	2464 (48.1)	2663 (51.9)		
	other	162 (2.0)	63 (38.9)	99 (61.1)		
had one or more children					4.693	0.030
	yes	4748 (59.1)	2259 (47.6)	2489 (52.4)		
	no	3282 (40.9)	1481 (45.1)	1801 (54.9)		
Profession-related characteristics						
professional qualifications					50.463	0.000
	diploma	3654 (45.5)	1859 (50.9)	1795 (49.1)		
	graduate	4240 (52.8)	1818 (42.9)	2422 (57.1)		
	postgraduate	136 (1.7)	63 (46.3)	73 (53.7)		
professional title					113.067	0.000
	junior	5542 (69.0)	2801 (50.5)	2741 (49.5)		
intermediate and senior		2488 (31.0)	939 (37.7)	1549 (62.3)		
administrative supervisor					122.711	0.000
	yes	918 (11.4)	270 (29.4)	648 (70.6)		
	no	7112 (88.6)	3470 (48.8)	3643 (51.2)		
received caring training in college					9.688	0.008
	yes	6748 (84.0)	3119 (46.2)	3629 (53.8)		
	no	869 (10.8)	398 (45.8)	471 (54.2)		
	not sure	413 (5.2)	223 (54.0)	190 (46.0)		
years in nursing profession		11.1 (8.5)	10.6 ± 8.4	11.6 ± 8.5	−5.258	0
years in present organization		9.1 (7.5)	8.6 ± 7.3	9.6 ± 7.7	−5.889	0
CDI *		107.5 (14.7)	106.4 ± 14.2	108.5 ± 15.0	−6.555	0
NCI *		6.0 (0.86)	5.9 ± 0.8	6.0 ± 0.9	−6.371	0
Workplace-related characteristics						
type of health facility					21.137	0.000
	hospital	7834 (97.6)	3617 (46.2)	4217 (53.8)		
	community	196 (2.4)	123 (62.8)	73 (37.2)		
workforce					0.159	0.924
	enough	2021 (25.0)	930 (46.2)	1082 (53.8)		
	barely enough	3146 (39.2)	1472 (46.8)	1674 (53.2)		
	not enough	2872 (35.8)	1338 (46.6)	1534 (53.4)		
provided caring training					39.031	0.000
	yes	5981 (74.5)	2664 (44.5)	3317 (55.5)		
	no	1298 (16.2)	684 (52.7)	614 (47.3)		
	not sure	751 (9.3)	392 (52.2)	359 (47.8)		

CDI, the caring dimensions inventory; NCI, the nurse’s career identity scale. * Result from mean (SD) and *t*-test.

**Table 2:** Predictors of willingness to participate in the fight against COVID-19 (multivariable logistic regression).

Variables	β	OR (95% CI)	*p*-Value
Socio-demographic characteristics			
gender		/	0.101
age		/	0.596
marital status			0.000
married vs. single ^R^	−0.33	0.72 (0.60–0.87)	0.000
other vs. single ^R^	0.03	1.03 (0.72–1.48)	0.878
had one or more children		0.016	
no vs. yes ^R^	−0.22	0.81 (0.68–0.97)	0.016
Profession-related characteristics			
professional qualifications	0.22	1.25 (1.14–1.37)	0.000
professional title	0.52	1.68 (1.50–1.90)	0.000
administrative supervisor			0.000
no vs. yes ^R^	−0.63	0.53 (0.45–0.63)	0.000
received caring training in college		0.153	
no vs. yes ^R^		/	0.285
not sure vs. yes ^R^		/	0.132
years in nursing profession		/	0.991
years in present organization		/	0.542
CDI	0.01	1.01 (1.01–1.01)	0.000
NCI		/	0.062
Workplace-related characteristics			
type of health facility			0.000
community vs. hospital ^R^	−0.64	0.53 (0.39–0.72)	0.000
human resource		/	0.496
provided caring training			0.000
no vs. yes ^R^	−0.26	0.77 (0.68–0.87)	0.000
not sure vs. yes ^R^	−0.21	0.81 (0.70–0.95)	0.009
Hosmer and Lemeshow test	chi-square value = 10.667		*p*-value = 0.221

CDI, the caring dimensions inventory; NCI, the nurse’s career identity scale. ^R^ = reference case; / = variable was excluded from the logistic regression model.

## Data Availability

All data that support the findings of this study are available from the corresponding author upon reasonable request.
